# The complete mitochondrial genome of *Macrosteles quadrilineatus* (Hemiptera: Cicadellidae)

**DOI:** 10.1080/23802359.2017.1303347

**Published:** 2017-03-23

**Authors:** Meng Mao, Xiushuai Yang, Gordon Bennett

**Affiliations:** Department of Plant and Environmental Protection Sciences, University of Hawaii, Manoa, Honolulu, USA

**Keywords:** *Macrosteles quadrilineatus*, mitochondrial genome, gene rearrangement, tandem repeats

## Abstract

The complete mitochondrial genome of the Aster leafhopper *Macrosteles quadrilineatus* was sequenced using an Illumina-based next-generation sequencing approach. The genome is 16,626 bp in length with 78.0% AT content. It encodes 37 typical mitochondrial genes including 13 protein-coding genes, 22 tRNA genes, 2 rRNA genes, and 1 A + T-rich region. Two tandem repeats were identified within the A + T-rich region. One tRNA gene rearrangement (*trnW*-*trnC*-*trnY→trnC*-*trnW*-*trnY*) was found between *nd2* and *cox1*.

The leafhopper family Cicadellidae (Hemiptera) is distributed worldwide with over 2600 genera and about 21,000 species (Camisao et al. 2014). The subfamily, Deltocephalinae, contains some of the most economically important leafhopper species, accounting for a disproportionate 77% of all agricultural pest species in the Cicadellidae (Zahniser & Dietrich 2008). One such species is the aster leafhopper, *Macrosteles quadrilineatus*, that is widespread throughout the North American continent. This species causes millions of dollars in agricultural and ornamental crop losses annually by vectoring the Aster Yellows phytoplasma that can infect hundreds of plant species (Hoy et al. 1992; Frost et al. 2011; Frost et al. 2013). *Macrosteles quadrilineatus* relies on bacterial symbionts for the provisioning of essential amino acids that are limited in their phloem diets, which also helped the host exploit novel niches (Moran 2007). The symbiont genomes of *M. quadrilineatus* were recently analyzed and revealed to be the smallest of any known bacterium (Bennett & Moran 2013). Despite the agricultural importance of *M. quadrilineatus*, population connectivity and species delimitation remains poorly understood, and research could benefit from additional molecular resources to address these questions.

Specimens of *M. quadrilineatus* were field collected from Yale West Campus, West Haven, CT, USA (GPS: 41°15′25.4″N 72°59′23.1″W) in 2013. Pinned representatives have been deposited in the University of Hawaii Mānoa Insect Museum (Accession Number: UHIM2017.00001, UHIM2017.00002 and UHIM2017.00003). 10 individual specimens were pooled for genomic DNA extraction with a Qiagen DNeasy kit. Library preparation and sequencing were done at the Yale Center for Genome Analysis. Genomic libraries were prepared from 500 base pair (bp) fragments and sequenced on an Illumina MiSeq (2 × 250 bp PE reads). Reads were *de novo* assembled with SPAdes V3.6.2 and contigs verified by assessing consistent read coverage by read mapping with Geneious v9.1.5 (Bankevich et al. 2012; Kearse et al. 2012). The completely assembled mitochondrial genome of *M. quadrilineatus* is 16,626 bp (GenBank no. KY645960) with an average read coverage of 1165×. Gene annotation was performed with Geneious v9.1.5 and further verified by comparison with the previously sequenced *Entylia carinata* mitochondrial genome (Mao et al. 2016). Finally, the 37 typical invertebrate mitochondrial genes (13 PCGs, 22 tRNAs, and 2 rRNAs) and the A + T-rich region were identified.

The A + T content of the *M. quadrilineatus* mitochondrial genome is 78.0%, which is similar to *E. carinata* (78.1%) (Mao et al. 2016). The conventional start codons ATA, ATG, and ATT could be assigned to 2, 4, and 7 PCGs, respectively. All of the 13 PCGs use the complete stop codons (*cox2* and *a6* use TAG, and the others use TAA). The putative A + T-rich region is 2141 bp long (84.1% A + T content) with two tandem repeats.

One tRNA gene rearrangement (*trnW*-*trnC*-*trnY→trnC*-*trnW*-*trnY*) between *nd2* and *cox1* was identified, when compared with the ancestral positions of other cicadellid species. This gene rearrangement has been commonly reported in other Hemipteran taxa (Wu et al. 2016; Zhou et al. 2016). The tandem duplication/random loss model is the most plausible mechanism to explain this local gene rearrangement (Boore 2000).

To verify the taxonomic and phylogenetic placement of *M. quadrilineatus*, we performed a maximum-likelihood phylogenetic analysis with RAxML (see Figure 1 for details). *M. quadrilineatus* formed a monophyletic group with other Cicadellidae species, which were recovered as the sister group of Membracidae. This is consistent with our previous analysis (Mao et al. 2016).

**Figure 1.**
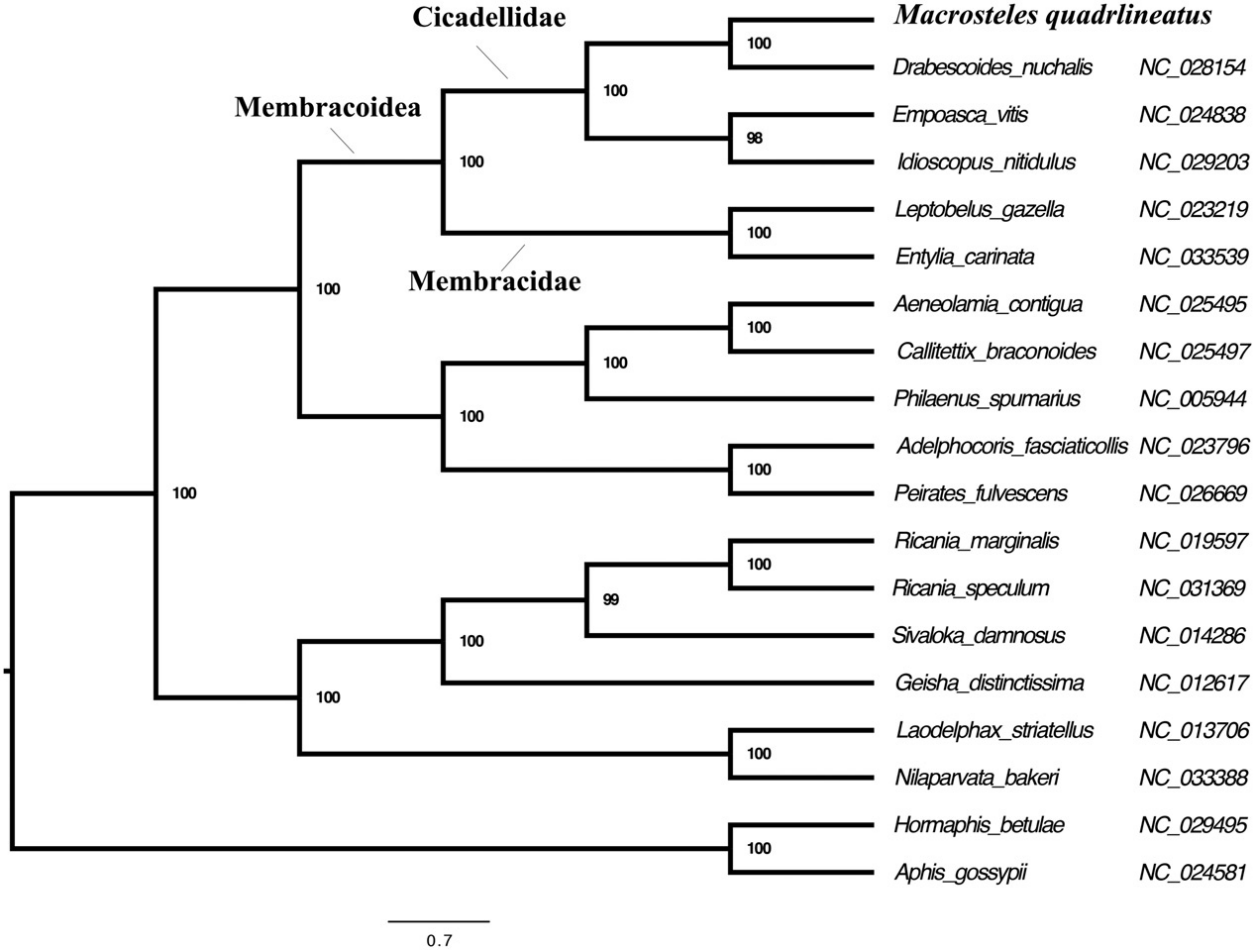
Maximum-likelihood phylogeny of Hemiptera species with fully sequenced mitochondrial genomes. Phylogenetic reconstruction was done from a concatenated matrix of 13 protein-coding mitochondrial genes with RAxML-HPC2 under the GTRCAT model in the CIPRES portal (Miller et al. 2010, Stamatakis 2006).
